# Composites with Re-Entrant Lattice: Effect of Filler on Auxetic Behaviour

**DOI:** 10.3390/polym15204076

**Published:** 2023-10-13

**Authors:** Mikhail Tashkinov, Anastasia Tarasova, Ilia Vindokurov, Vadim V. Silberschmidt

**Affiliations:** 1 Laboratory of Mechanics of Biocompatible Materials and Devices, Perm National Research Polytechnic University, Komsomolsky Ave., 29, 614990 Perm, Russia; 2 Wolfson School of Mechanical, Electrical and Manufacturing Engineering, Loughborough University, Leicestershire LE11 3TU, UK

**Keywords:** metamaterials, negative Poisson’s ratio, auxetics, re-entrant unit-cell, lattice structure, failure probability, composite structures

## Abstract

This study is focused on the deformation behaviour of composites formed by auxetic lattice structures acting as a matrix based on the re-entrant unit-cell geometry with a soft filler, motivated by biomedical applications. Three-dimensional models of two types of the auxetic-lattice structures were manufactured using filament deposition modelling. Numerical finite-element models were developed for computational analysis of the effect of the filler with different mechanical properties on the effective Poisson’s ratio and mechanical behaviour of such composites. Tensile tests of 3D-printed auxetic samples were performed with strain measurements using digital image correlation. The use of the filler phase with various elastic moduli resulted in positive, negative, and close-to-zero effective Poisson’s ratios. Two approaches for numerical measurement of the Poisson’s ratio were used. The failure probability of the two-phase composites with auxetic structure depending on the filler stiffness was investigated by assessing statistical distributions of stresses in the finite-elements models.

## 1. Introduction

Architected materials and structures have attracted the attention of researchers as a novel design and engineering concept in aerospace [1], automotive [2], sports and biomedical industries [3]. Recent advances in additive-manufacturing (AM) methods have enabled the production of complex three-dimensional structures with tailored properties, combining their light weight with high impact resistance, compressive strength and damping abilities [4]. One of the most promising advantages of topological freedom, provided via additive manufacturing, is the ability to create metamaterials—a class of materials and structures that are engineered to exhibit properties that are unattainable in natural or traditionally manufactured analogues. One such specific mechanical property is a negative Poisson’s ratio (NPR). Materials with an NPR behaviour, known as auxetics, expand laterally under longitudinal tension and contract under compression [5,6]. Such a deformation mechanism is beneficial in achieve high impact strength, shear, indentation and fracture resistance. For these reasons, auxetic metamaterials have great potential in many fields, particularly in the aviation industry [7], sports applications [8], electronics [9] and biomedical engineering [10]. Focusing on the last of these fields, the advantages of auxetic metamaterials can be used to produce various biomedical devices with improved performance. For instance, structures with a combination of auxetic and non-auxetic behaviour can be used in hip implants. The auxetic behaviour can improve the contact between the bone and the implant. In [1], a numerical calculation of free vibration of structures including a combination of them with negative and positive Poisson’s ratios was performed. In [11], the authors investigated the compressive mechanical properties of titanium alloy specimens based on a re-entrant hexagonal cells printed using additive technologies. The fatigue behaviour of these structures as well as crack initiation and propagation mechanisms was also investigated, as the implants have a problem of loosening under cyclic loading [12,13]. Additionally, auxetic structures are implemented in the design of novel types of stents and orthopaedic implants [14].

Mechanical metamaterials and, particularly, auxetics gain extraordinary effective properties from rational design and architecture of their microstructure [15,16,17,18]. Many of them have a lattice structure and are created via the duplication of a specifically designed unit cell in multiple directions. A microstructure of auxetic media is usually based on different unit-cell geometries and mechanisms: re-entrant hexagonal unit-cells [19]; star-shaped inclusions [20]; chiral configurations [21,22]; perforations and cuttings [23,24]; rotating rigid units [25,26] and others. 

Different parameters of auxetic structures can be achieved via the variation of the re-entrant hexagonal unit-cell’s angle gradient [27,28] and rib length [29]. A novel design of the re-entrant circular auxetic honeycomb, suggested in [6], demonstrated a more pronounced NPR effect at the earlier deformation stages. By combining the re-entrant NPR structure and a hexagonal structure with a positive Poisson’s ratio, a type of mechanical metamaterial was developed that expands transversely regardless of the sign of uniaxial loading [30].

Chiral auxetic grids based on shape-memory alloys were numerically investigated in terms of their tensile strength and used for noise and vibration suppression [31]. Three-dimensional lattice structures (with negative and positive Poisson’s ratios), employing a stretching-twisting effect of a chiral lattice structure, were also considered. Numerical calculations and compression experiments were carried out, and a wide range of Poisson’s ratios was obtained [15,32]. Tensile properties of auxetics with star-shaped perforations, which can reach negative and zero Poisson’s ratios, was analysed numerically and experimentally using 3D printing [20]. They maintained their properties at strains greater than 15%. Hierarchical structures based on perforations with six-fold symmetry, which ensures an isotropic medium, assessed numerically, were confirmed with a uniaxial tension experiment [23]. 

Auxetics were also reported to be used in multi-material structures, such as filled cellular structures [33,34] or tubes with various fillers [35,36,37,38]. Composites, with a matrix phase in a form of auxetic-lattice, showed enhanced compressive strength and energy absorption under compression. The auxetic matrix can be either stiffer or softer than the filler. The latter is named the brick-and-mortar composite in which the soft matrix behaves as the mortar and the filler corresponds to periodically arranged bricks [34]. Additionally, for example, a polymer-filled aluminium auxetic demonstrated better properties than its auxetic-lattice [35]. A three-component composite was designed and fabricated by filling polymer into the auxetic-lattice structure as an inner part and adding an empty tube as a restraint boundary [39]. There have also been studies of auxetic honeycombs [37], auxetic foam [40,41,42], double-arrowed [38] and gradient auxetic structures [36], as well as printed polymer chiral structures filled with foam [43] and a circular auxetic tube with elliptical holes filled with foam [44].

Despite the fact that some research addressing theoretical and practical question of the auxetic composites was performed, there is still a need for a fundamental understanding of the filler’s effect on the auxetic properties of the composites with auxetic-lattice. This can be useful, for instance, for design of biomedical implants and devices, to predict their behaviour affected by the surrounding media and changes to their auxetic performance (in terms of NPR).

In this research, an auxetic-lattice structure and two-phase composites with this auxetic matrix and a filler of different properties were considered. The structures were designed based on the re-entrant unit-cell with axial and transverse orientations. The re-entrant auxetic-lattice was manufactured with fused deposition modelling/fused filament fabrication (FDM/FFF) and tested under tension, with its surface strain field captured with the digital image correlation (DIC) technique. Finite-element (FE) models of the two-phase auxetic composites with various elastic properties of the filler were developed. A comparison of numerical and experimental results for the strain fields was performed for verification of the porous-lattice models. The effect of the elastic modulus of the filler on the effective Poisson’s ratio of two-phase auxetic composite structure was examined. 

## 2. Materials and Methods

### 2.1. Design of Auxetic Structures

**Figure 1:**
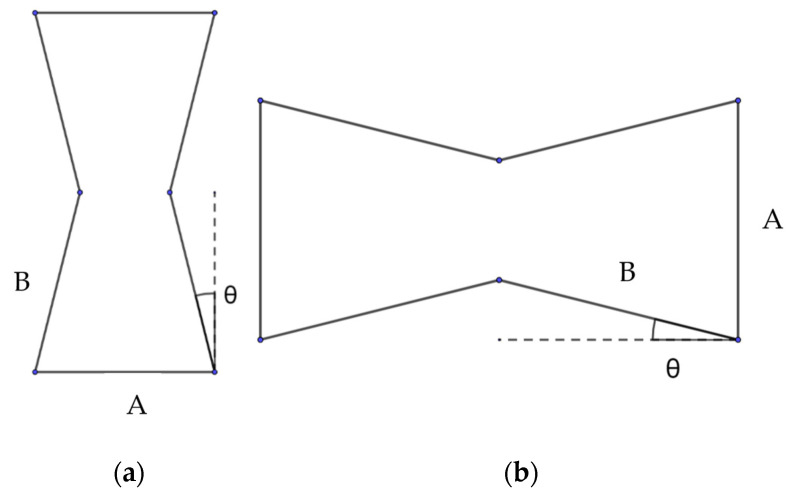
Dimensions of auxetic unit-cell: (**a**) axial and (**b**) transversal orientations.

Among the auxetic cells with complex geometry, a re-entrant hexagonal honeycomb is one of the simplest patterns that exhibit the NPR effect [33]. The geometry of its unit-cell allows the structure to expand laterally when a tensile load is applied. This behaviour is retained regardless of the orientation of the unit-cell (see Figure 1a,b). The resulting NPR depends on several geometrical parameters, such as the re-entrant angle (θ) and the length ratio of ribs (a/b) [11].

**Figure 2:**
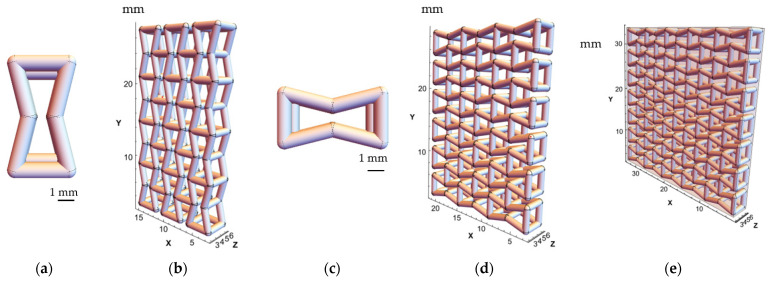
(**a**) Model of axially oriented re-entrant unit-cell; (**b**) axially oriented porous auxetic-lattice structure; (**c**) model of transversely oriented re-entrant unit-cell; (**d**) transversely oriented porous auxetic-lattice structure; (**e**) lattice of two-phase auxetic structure.

A three-dimensional model of the unit-cell geometry was developed with two simple entities: cylinders were used as struts and spheres as joints to connect structural elements and provide a smooth transition between them. The following combination of parameters was used to create structures with both orientations of the unit-cell: A/B=1, θ=14° (Figure 1). The cylinder elements (struts) and spheres (joints) had the radius of 0.3 mm (Figure 2a,c).

Samples with two types of structural compositions were studied in this work. The structures of the first group were porous and produced with the aim to compare the results of FE modelling with experimental assessment of strain fields, using the DIC system during tension loading of the additively manufactured (AM) samples. In the structures of the second group the same lattices contained the second-phase (filler) and were used to investigate the second-phase influence with numerical simulations.

For the samples of the first group, the structures with the axial orientation were created by repeating the axially oriented unit-cell (Figure 1a and Figure 2a) three times along the *X* axis and four times along the *Y* axis to achieve the overall dimensions of 12.9 mm × 24.9 mm × 3.9 mm (Figure 2b). To obtain the transversely oriented structure, the unit-cell (Figure 1b and Figure 2c) was repeated 3 times along the *X* axis and 6 times along the *Y* axis, resulting in the overall dimensions of 18.9 mm × 26.4 mm × 3.9 mm (Figure 2b,d).

The two-phase structures (second group) were composed of 7 × 5 × 1 axially oriented unit-cells; dimensions of the resulting structure were 30.9 mm × 30.9 mm × 3.9 mm. As a result, the structured formed a square in the XY plane, allowing the use of the same structure for analysis of both axial and transversely oriented cases by performing its 90-degree rotation along the *Z* axis (Figure 2e).

### 2.2. Additive Manufacturing and Mechanical Testing of Auxetic Structures

**Figure 3:**
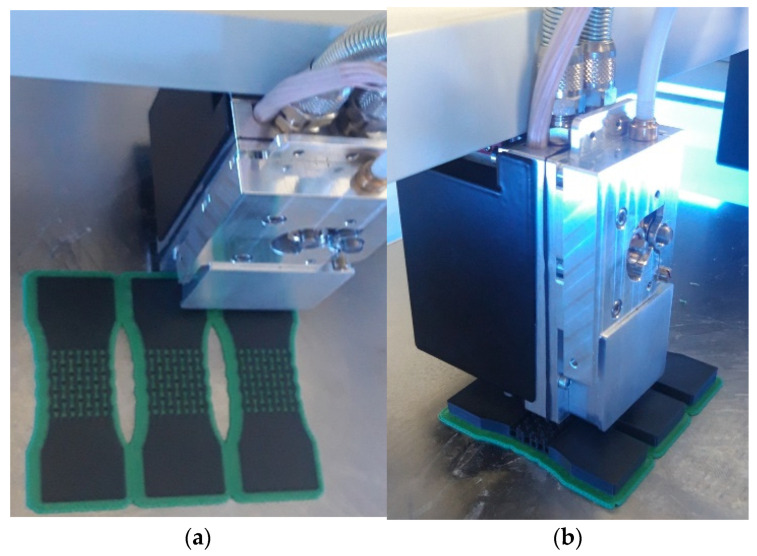
Additive manufacturing of specimens: (**a**,**b**) 3D printing process.

The samples of the first type were manufactured from the high-impact polystyrene (HIPS) using the FDM/FFF AM technique [23]. The models were printed with a 0.4 mm diameter nozzle at a speed of 1800 mm/min using F2 Lite printer by F2 Innovations (Perm, Russia). The height of the first layer was 0.2 mm followed by further layers with 0.1 mm height. The temperatures of the table and the nozzle were 110 °C and 255 °C, respectively. A raft (horizontal grid of filament underneath the model) was used to discard the effects of the table curvature and to increase the level of adhesion. Additionally, the rafts are used as a solid base for the first layers of the model if the contact area of the model with the table is too small. Three specimens were produced for each of geometry configuration—axial and transversal (Figure 3). The specimens were manufactured with end tabs to support mechanical tests.

Tensile tests were conducted for the specimens from the first group. A universal mechanical testing machine Instron (Glenview, IL, USA) 68SC-5 with a 500 N load cell was used. Specimens were mounted vertically by uniformly clamping their upper and lower edges and then subjected to tensile load of 130 N for the axially oriented samples and 38 N for the transversely oriented ones, with a speed of 1 mm/min.

### 2.3. Digital Image Correlation

**Figure 4:**
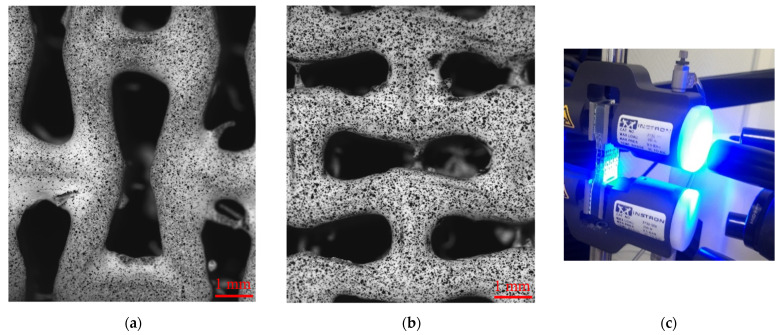
(**a**) Axially oriented unit-cell of AM sample with applied speckles; (**b**) transversely oriented unit-cell of AM sample with applied speckles; (**c**) tensile test of sample with Vic-3D Micro-DIC system.

Distribution of displacements and strain fields on the surface of the samples were registered with the DIC technique. By comparing displacements of a predefined texture on a surface of a specimen during its deformation, this method provides a non-destructive and non-contact approach to study the deformation behaviour of materials and structures [45]. To create a contrast image texture on a sample surface, a black-and-white random speckle pattern was applied. The surface of the manufactured specimens was painted in white, while black speckles were eventually added using an airbrush (Figure 4a,b). An isi-sys Vic-3D Micro-DIC system (Correlated Solutions, Irmo, SC, USA) was employed in this study. Two 5 MP digital cameras were used to capture the front surface of the specimens with a frequency of 1 Hz. Once the cameras were set, the DIC system was calibrated using the VicSnap 9 software. The obtained during the experiment set of images was analysed using Vic-3D 9 to calculate the displacements and strain fields [22]. The field view of Vic-3D Micro-DIC system was 8.4 mm × 7 mm, allowing detailed images of a single unit-cell (see Figure 4).

### 2.4. Finite-Element Analysis

Three-dimensional FE models were developed to investigate the mechanical behaviour of both lattice and two-phase auxetic structures under tensile load. Each structure was discretized using tetrahedral solid elements in Wolfram Mathematica (Champaign, IL, USA) and then transferred using a developed script to SIMULIA Abaqus (Dassault Systemes, Montréal, QC, Canada) as a model with tetrahedral C3D4 elements type (continuum three-dimensional with four nodes) to perform quasi-static tension simulations. The corresponding material constants and section properties were assigned by the script. 

Discretisation of the two-phase composite structures requires a continuous interphase between the phases. Applying methods of traditional discretisation is not efficient in this case because of a large number of lattice elements that need to be meshed and coupled—the lattice itself was formed with complex Boolean-type compositions, such as intersecting cylinders (struts) and spheres (joints). So, entities of a complex shape, belonging to the second-phase, should be formed by difference operation performed on a lattice and a bounding parallelepiped, which sides limit the lattice. 

The Open CASCADE meshing technique, implemented in Wolfram Mathematica, was tested for discretisation of porous lattice structures. It allows two-dimensional triangulation of surfaces of the Boolean-type structures, which can be subsequently used as a basis for tetrahedral solid internal meshing [46]. However, this method was found to be computationally expensive for the considered cases—both in terms of discretisation and the resulting number of finite elements, which could lead to further computational difficulties in numerical analysis of the mechanical problems. Besides, the entities generated with the difference Boolean operation, required for the second-phase, sometimes cannot be discretised with this algorithm.

A solution was found in the following two-step discretisation procedure. At the first step, both phases were discretised with a voxel-based regular mesh, commonly used for FE analysis of structures with non-trivial geometry. The general drawback of a voxel mesh is presence of sharp stepped edges of a border due to the cubic form of individual voxels. To obtain tetrahedral discretisation with a smooth surface, the Dual Marching Cubes algorithm, implemented in Wolfram Mathematica, was applied to the voxel mesh of both phases at the second step. This iterative computational algorithm was designed to generate smooth separating surfaces for binary, enumerated volumes, often produced with segmentation algorithms [47,48]. Two meshes, that form a two-phase structure, were then merged by combining nodes on the interface. In the case of single-phase lattice structures, the filler phase can be omitted.

**Figure 5:**
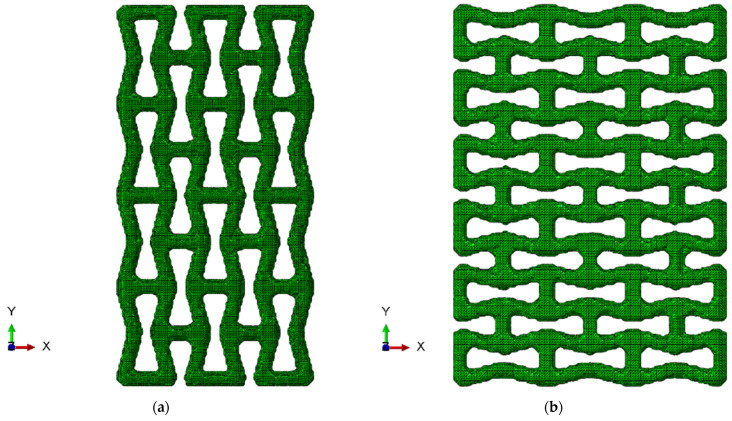
Axial (**a**) and transversal (**b**) orientations of lattice.

The obtained discretised models of the structures, that were presented in Figure 2, are shown in Figure 5. This method generates dense FE models for the structures from the first group, with a total number of approximately 400,000 tetrahedral elements with an average size of 0.005 mm.

**Figure 6:**
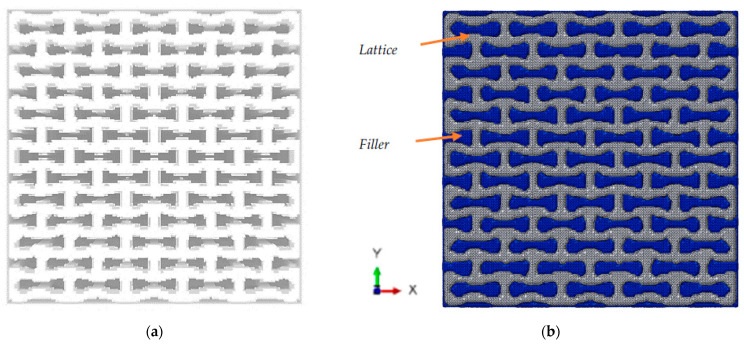
Voxel (**a**) and tetrahedral (**b**) FE models of two-phase auxetic structure.

The discretised two-phase model of the filled lattice structure is presented in Figure 6. It consists of approximately 700,000 tetrahedral elements: 300,000 elements in the first phase, and 400,000 elements in the second (filler). Computations were performed using SIMULIA Abaqus 2022 Standard.

The lattice structure was modelled as HIPS, which isotropic properties were supplied by the manufacturer: elastic modulus of 2000 MPa, Poisson’s ratio of 0.35 and density of 1.09 g/cm^3^.

The varying model properties were assigned to the second (filler) phase in order to study their effect on the mechanical behaviour and the Poisson’s ratio of the lattice structure. The values of the used elastic modulus of the filler phase and the ratios between the elastic moduli of the two-phases are shown in Table 1. The low values of this parameter are motivated by biomedical applications, e.g., polymeric auxetic scaffolds surrounded by soft biological tissues.

A tensile displacement along the *Y* axis was applied to the top nodes of each structure in both groups, while their other degrees of freedom were constrained. The bottom nodes were constrained in all directions. The range of modelled displacement values corresponded to 0.25%, 1%, and 3% of strain.

The effective Poisson’s ratio of the structures was calculated via two approaches: by global averaging of strain values in mesh elements and by measuring displacements of the specified nodes located in the predetermined unit-cells (as in mechanical tests). The standard relation for the Poisson’s ration was used: $\nu ={-\varepsilon }_{11}/\varepsilon_{22}$, where $\varepsilon_{11}$ is the lateral deformation caused by the applied axial deformation $\varepsilon_{22}$.

In the first case the mean values of strains were calculated by weighted averaging of strains in all finite elements existing in the mesh. Since in the tetrahedral mesh every element has its own different volume, the strain value in each element was taken into account with a corresponding to its volume statistical weight. The average strain tensor was obtained after processing the ODB database, obtained after computations with SIMULIA Abaqus, in MSC Digimat 2017 software.

In the second case, the lateral strain $\varepsilon_{11}$, required for the Poisson’s ratio estimation, was measured as deformation of a line, parallel to the *X* axis, connecting two opposite points at the edges of the lattice. A number of such lines depends on a number of unit-cells repeated along the *Y* axis (11 in case of axially oriented and 13 in case of transversely oriented structures). Relative extension or contraction of these lines define the lateral strain $\varepsilon_{11}$. The value of the global axial strain $\varepsilon_{22}$ was determined from the boundary conditions.

### 2.5. Statistical Analysis

Critical values of the stress fields may not be explicitly observed if they are inside the structural elements. In this work, the statistical approach was applied to the results of FE simulations to analyse stress fields presented as statistical distributions of random variables. Knowing the value of stress field in each element as well as its volume, it is possible to create histograms of probability to find the stress value in the predetermined range [49,50,51,52]. Hence, the histogram bars indicate probability (in the range 0 to 1) that depends on the value of the stress field [53]. They allow to evaluate the relative volume of the model, with stresses (or stress invariants) exceeding some thresholds. For instance, in case when such a threshold is a critical maximal stress value, the bars beyond the threshold correspond to the failure probability of the model.

This technique was also applied to analyse of the two-phase auxetic structures. In order to compare structures with different filler properties, the maximal principal stress values normalised with the value of the tensile strength of the lattice material was used as the dimensionless stress measure.

## 3. Results

### 3.1. FE Simulations of Porous Lattice Structure

**Figure 7:**
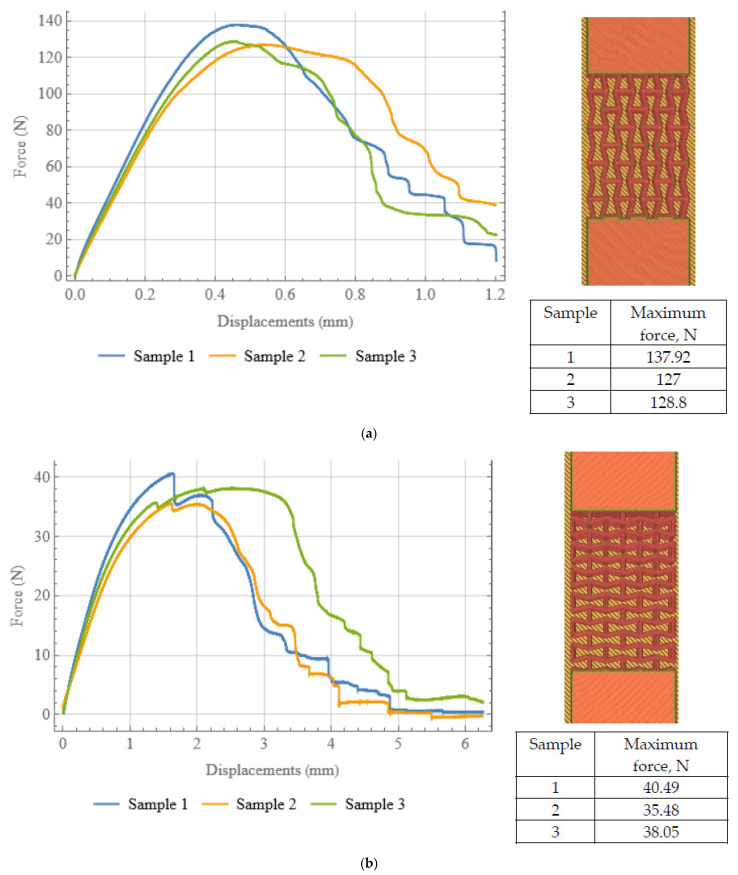
Tensile force vs. displacement diagram (loading curve) recorded in tensile tests for axially (**a**) and transversally (**b**) oriented samples.

The experimentally measured displacements–force curves for samples of each orientation are presented on Figure 7. The variability of the experimental data is typical for specimens produced with additive manufacturing.

For the axially oriented samples (Figure 7a) no significant spikes on the curves are observed, since this type of structure has more joints than the transversely oriented structure. On the contrary, the curves for the transversely oriented samples (Figure 7b) have surges, which correspond to ruptures in structural joints, which lead to further redistribution of load, followed by subsequent gradual failure.

**Figure 8:**
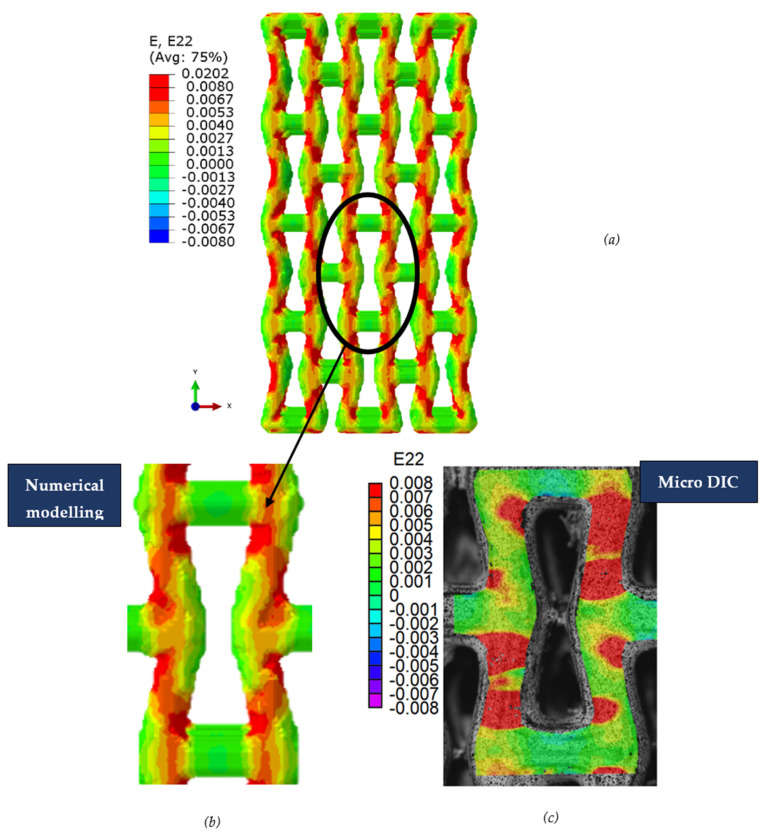
(**a**) Field of $\varepsilon_{22}$ strain for axial direction auxetic-lattice. Data for cell obtained numerically (**b**) and experimentally (**c**).

**Figure 9:**
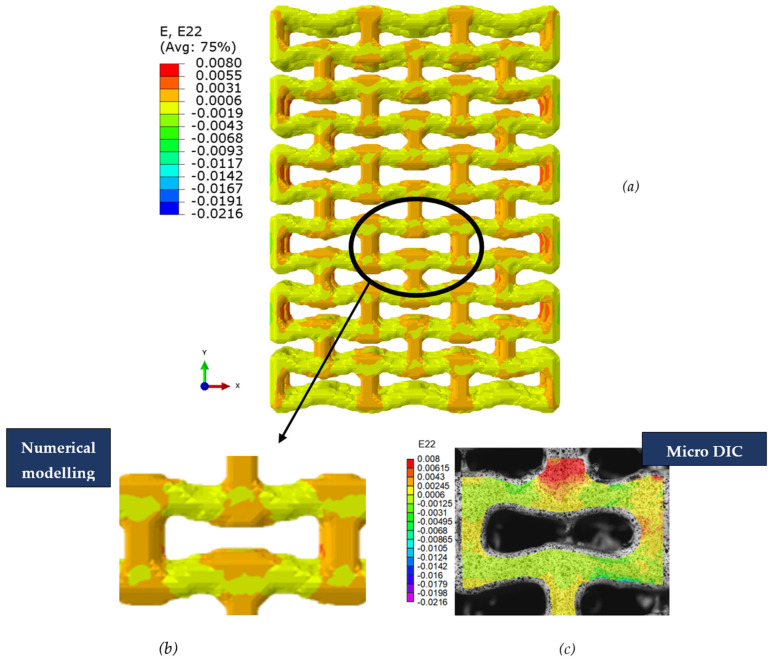
(**a**) Field of $\varepsilon_{22}$ strain for transversal direction auxetic-lattice. Data for cell obtained numerically (**b**) and experimentally (**c**).

Distributions of strain field $\varepsilon_{22}$ for porous lattice structures were obtained numerically from three-dimensional FE models as well from DIC experimental results. They are compared on Figure 8 and Figure 9 for both axial and transverse orientation of the lattice. Distributions of numerically calculated strains are presented for the whole model. The unit-cell in the model, which was assessed with DIC during the experiments, was zoomed in for direct comparison with the experimentally obtained distribution; the same colour scale was used.

The strain patterns observed in the experiments are in a good agreement with those predicted by the numerical simulations. The highest values (orange and red zones) of $\varepsilon_{22}$ were found at the inclined struts and at the connection points of the axially oriented geometrical features (Figure 8 and Figure 9). For a structure with transversal orientation of unit-cells, the maximum strain values were located in the struts connection and in axially oriented structs (Figure 9). Strain fields were different for structures with axial and transversal orientations since inclined struts of the re-entrant hexagonal honeycomb unit-cell are more prone to tensile displacements than vertical ones. Some inconsistencies and differences between the experimental data and the numerical results for the strain fields are naturally explained by manufacturing-induced defects, occurring during the FDM/FFF 3D-printing process. The nature of this technology, based on the layer-by-layer filament placement, eventually leads to imperfections at the interfaces between the layers. These defects result in uneven (non-smooth) lateral surfaces [54], affecting the images captured with the DIC technique.

The obtained results demonstrate that the developed three-dimensional FE models correctly simulate the mechanical behaviour of the auxetic-lattice structures during tensile loading. This allows further numerical investigations focused on the effect of the second-phase on the auxetic properties of the structures. A full spectrum of properties variation of the filler phase is cumbersome to implement in experimental studies due to a lack of materials, suitable for AM, which elastic properties can be gradually changed and controlled. The latter is important to establish the combinations of the phase properties to fine-tune the mechanical response of the two-phase composite structure from auxetic, with a zero-level Poisson’s ratio, or with a positive Poisson’s ratio. Thus, this task may be resolved numerically.

### 3.2. Effect of Filler on NPR

In order to study the influence of the filler on auxetic properties of the lattice structure, different values of the elastic modulus (according to Table 1) were assigned to the filler phase. The obtained numerical results demonstrated that mechanical behaviour of the whole structure, and, specifically, its auxetic properties, depended on these properties and, therefore, can be controlled. This effect can be explicitly observed by comparing the field of lateral displacement $u_{1}$ for the structures with both axial and transversal orientations and different filler stiffness subjected to the same 0.25% tensile strain along the *Y* axis. Table 2 shows the changes in displacements field u_1_ for the axially and transversely oriented two-phase structures with filler’s modulus starting from 600 MPa to 0.2 MPa (ratio of filler modulus to modulus of lattice $E_{filler}/E_{lattice}$ varies from 0.3 to 10^−4^) and porous structure. The gradient of displacements fields reflects the behaviour of the structure: with the *X* axis directed to the right, negative values (blue colour) on the right and positive values (red colour) on the left correspond to lateral contraction of the structure, i.e., positive Poisson’s ratios. This behaviour is observed for the structures with elastic modulus equal to 600 MPa, 200 MPa and 60 MPa $(E_{filler}/E_{lattice}$ equal to 0.3, 0.1 and 0.03). On the contrary, positive values on the right and negative values on the left indicate lateral expansion (negative Poisson’s ratio), i.e., auxetic behaviour. The structures from the considered cases with elastic modulus of 2 MPa, 0.2 MPa and 0 MPa (porous lattice) ${(E}_{filler}/E_{lattice}\;$ equal to $10^{-3}$, $10^{-4}$ and 0) fall in that category. The chosen tensile displacement values allowed us to see this tendency for the two-phase structure with both axial and transverse orientations of unit-cells (see Table 2). When a filler’s elastic modulus is 20 MPa ${(E}_{filler}/E_{lattice}=0.01)$, both axially and transversely oriented structures demonstrate a close to zero value of Poisson’s ratio.

Besides, different levels of tensile strains applied to the two-phase structures were considered in simulations: 0.25%, 1.5% and 3%. The results obtained with for these values show somewhat similar patterns for axial and transversal directions of the auxetic-lattice geometry.

**Figure 10:**
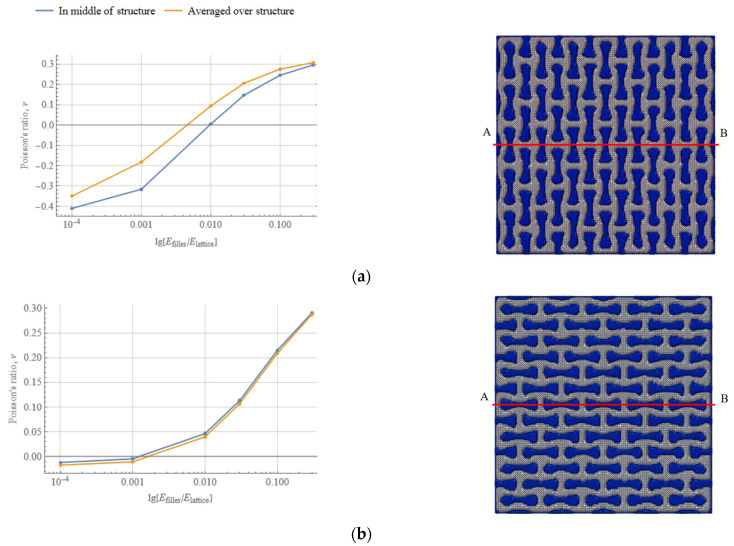
Dependence of Poisson’s ratio on lg[$E_{filler}/E_{lattice}$] for axially (**a**) and transversally (**b**) oriented re-entrant auxetic structures.

The important question is about the relative value of the filler’s elastic modulus (compared to that of the lattice) that reverses the effect of the auxetic re-entrant design. In order to establish it, the change of global Poisson’s ratio was presented in dependence on the logarithm of the ratio $E_{filler}/E_{lattice}$. The results for the two studied orientations of the structure are presented on Figure 10. Two curves for the Poisson’s ratio values were obtained using the two above-described approaches: averaging over the structure and measured in its middle (line AB in Figure 10). The greater the value of the elastic modulus of the filler, the higher is the value of the global Poisson’s ratio along the *Y* axis. For instance, when the elastic modulus of the filler reaches 200 MPa ${(E}_{filler}/E_{lattice}=0.1)$, the overall Poisson’s ratio is about 0.24 for the axial orientation and 0.22 for the transverse one; when the filler’s modulus is reduced down to 0.2 MPa ${(E}_{filler}/E_{lattice}=10^{-4})$, the Poisson’s ratio of the structure is about −0.41 for the axial orientation, and is close to 0 for the transverse orientation, as measured in the middle of the structure.

The values of the Poisson’s ratio measured with two discussed methods do not coincide for the axially oriented structure. This can be explained by the location of the points used for measurements: geometrical composition of the axially oriented structure leaves the filler phase on the right and left sides in the middle of the *Y* axis. This filler surplus has its influence on the local parts of the lattice.

Plots in Table 3 and Table 4 show distributions of $\varepsilon_{11}$ strain component for the two-phase structures. Blue and red lines connect the pairs of points for which the Poisson’s ratio was measured; the former originate and end in the lattice, the latter—in the filler. According to the evenly selected distance, the odd-numbered lines connect points at the edge of the lattice, and the even-numbered lines begin and end in the filler. Apparently, the red-lines measurements are more influenced by the filler: its volume fraction for these lines is larger than the blue ones. The colour of dots in the graphs for the Poisson’s ratio corresponds to that of the respective line. The axis of abscissas gives the line number from the top to the bottom of the structure. The green line demonstrates the value of the global (averaged over the two-phase structure) Poisson’s ratio.

Evidently, the obtained values of the Poisson’s ratio are highly influenced by the type of the specified boundary conditions: restrictions imposed on the top and the bottom of the composite structure do not allow its lateral expansion, leading to a barrel-like deformed shape. This was done in order to simulate tension experiments on the real additively manufactured samples. As a result, the values of the Poisson’s ratio, measured for the rows closer to structure’s boundaries along the *Y* axis, were higher than those in the middle. The lowest value of the Poisson’s ratio, and, consequently, the most pronounced auxetic behaviour was reached in the middle of the models.

Table 3 shows distribution of the Poisson’s ratio for the axially oriented structures with the filler’s elastic modulus of 200 MPa, 20 Mpa and 0.2 Mpa (*E_filler_*/*E_lattice_* = 0.1, 0.01, 10^−4^). All the measured values and the mean value of the Poisson’s ratio are confidently in the positive zone for the filler with elastic modulus of 200 Mpa (*E_filler_*/*E_lattice_* = 0.1). When the modulus of the filler decreased 10-fold, down to 20 Mpa (*E_filler_*/*E_lattice_* = 0.01), the Poisson’s ratio for one of the measured pair of points (line 6) falls below 0, while other points—as well as the globally measured value—were still positive. This marks the threshold for the elastic properties of the filler corresponding to the transition from the negative to positive values of the overall Poisson’s ratio, i.e., loss of the auxetic behaviour. The filler with the elastic modulus of 0.2 Mpa (10,000 times lower than the modulus of the lattice, *E_filler_*/*E_lattice_* = 10^−4^) did not affect the designed auxetic properties of the lattice.

Table 4 presents the similar results for the transversely oriented two-phase structure. As in the case of the axially oriented structure, all presented values of the structural Poisson’s ratio are in the positive zone for the filler with the elastic modulus of 200 Mpa (*E_filler_*/*E_lattice_* = 0.1). For the case of 2 Mpa (*E_filler_*/*E_lattice_* = 10^−3^), the average value of the Poisson’s ratio is negative, while there are some locally measured values demonstrating the opposite behaviour. This can be explained by proximity of the measured points to the constrained surfaces. The average values of the Poisson’s ratio also indicated the transition point for the elastic properties of the filler that divides the auxetic and the non-auxetic behaviours of the filled lattice. The filler with the lowest elastic modulus of 0.2 Mpa (*E_filler_*/*E_lattice_* = 10^−4^) embedded into the transversally oriented structure had a value much closer to the zero value of the global Poisson’s ratio than the axially oriented structure.

The mean averaged over the structure value of the Poisson’s ratio generally was found to be somewhere between the locally measured values. The stiffer the filler, the more influence it had on the averaged value. The Poisson’s ratio measured for the blue lines was always higher than that for the red lines due to the contrast of the properties and local variations of the volume fractions. This tendency can be clearly observed in the plots in Table 3 and Table 4. For instance, for the axially oriented structure with minimal filler stiffness, the mean value of the Poisson’s ratio is close to the average value of the ratio measured for the discrete local points. As a result of the increasing filler stiffness, the average Poisson’s ratio green line moves closer towards the values measured for the lines with higher filler concentration (red dots). The same pattern was observed for the transversely oriented structure. However, in the latter case, the mean value of the Poisson’s ratio for the stiffest filler became even lower than the values in the measured pair of points. This can be explained by the growing influence of the lateral strain values in the internal parts of the structure. The filler exerted an effect on the lattice, preventing its lateral expansion.

For the structure with an axial orientation of unit-cells, a filler with stiffness that is only 1% of that of the lattice can hinder auxeticity of the lattice design: the Poisson’s ratio is close to (and slightly above) zero. An increase in filler stiffness up to 10% of the lattice is level changed the structure’s behaviour to a standard mechanical response, with a positive Poisson’s ratio. Thus, the axially oriented re-entrant type lattice retains its auxetic behaviour only with low stiffness properties of the filler: when the elastic modulus of filler is about 0.01% of the that of the lattice material, the auxetic properties are preserved.

Auxetic properties of the structure with transverse orientation of the unit-cells are initially less pronounced than for the structure with axial orientation of the unit-cells. This confirms the established fact that the geometry of the unit-cell is an important parameter that can be controlled to tailor auxetic properties of the structure. For this type of geometry, the zero-value Poisson’s ratio of the structure was reached when the elastic modulus of the filler was only 0.1% of the lattice’s modulus. The stiffer filler would certainly increase the global Poisson’s ratio and, consequently, diminishing the structural auxeticity. 

### 3.3. Analysis of Stress Distributions in Lattice

**Figure 11:**
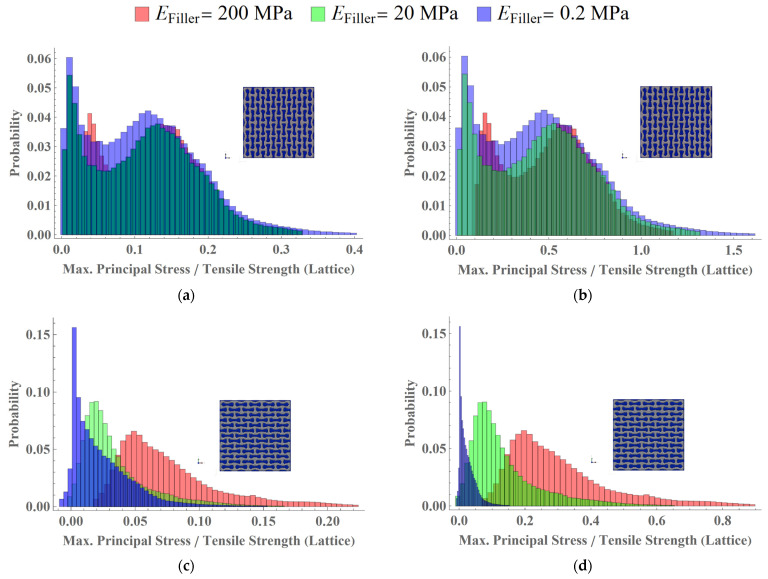
Comparison of distribution of normalised maximum principal stress (with tensile strength) fields for axial (**a**,**b**) and transversal (**c**,**d**) two-phase structures with filler elastic modulus of 200, 20 and 0.2 MPa ($E_{filler}/E_{lattice}=0.1,\;0.01\;and\;10^{-4}$): for various applied strains: (**a**,**c**) 0.25%; (**b**,**d**) 1%.

The simulations also demonstrated the effect of filler on mechanical performance of two-phase composites with auxetic lattices. Histograms of stress distributions in the lattice for the axially and transversely oriented structures under induced applied strains of 0.25% and 1% are presented on Figure 11. Three cases of filler elastic properties were considered: 200 Mpa, 20 Mpa and 0.2 Mpa (*E_filler_*/*E_lattice_* = 0.1, 0.01 *and* 10^−4^). The tensile strength of the lattice material (HIPS) was measured with in-house experiments and was taken as 30 Mpa.

For the tensile strain of 0.25%, the normalised maximal principal stress is between 0 and 1 for all the elements of the lattice, in cases of all fillers. This means that the strength criterion was not reached, and failure process did not start. When the tensile strain reached 1% of strain (Figure 11b), the histograms’ tails move to the right, exceeding the value of 1 for some cases. This means that in some parts of the structures, the failure criterion was satisfied.

The obtained results show how the filler properties can be used to control the damage behaviour of the two-phase structure. More rigid filler could prevent stress growth in the lattice, while structures with more pliable filler accumulate critical stress faster.

## 4. Conclusions

The mechanical behaviour of the two-phase composite structures, consisting of the auxetic-lattice and the softer filler with variable properties has been evaluated. Numerical models were developed to simulate the mechanical behaviour, and stress distributions in the two-phase structures as well as to calculate the resulting Poisson’s ratio. The effect of the variation of filler’s elastic modulus on the Poisson’s ratio of the structures was also examined. It was demonstrated that the change in the elasticity properties of the filler could dramatically reduce the ability of the structure to retain the negative value of the Poisson’s ratio. This effect could be used to tailor and predict the behaviour of the auxetic-lattice structures that are designed to be used in contact with the surrounding media, for example, in biomedical applications.

## Figures and Tables

**Table 1:** Characteristics of lattice and two-phase structures.

Elastic Modulus of Auxetic-Lattice, $E_{lattice}$, Mpa	Elastic Modulus of Filler, $E_{filler}$, MPa	Ratio between Elastic Moduli, $E_{filler}/E_{lattice}$
2000	600	0.3
	200	0.1
	60	0.03
	20	0.01
	2	0.001
	0.2	0.0001

**Table 2:** Fields of displacement u_1_ (along *X* axis) for various fillers.

Elastic modulus of filler $E_{filler}$, MPa	600	200	60	20	2	0.2	0
Relation between elastic moduli $(E_{filler}/E_{lattice}=0.01)$	0.3	0.1	0.03	0.01	0.001	0.0001	0
Axial orientation of structure	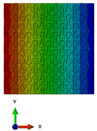	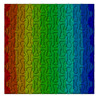	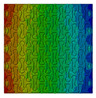	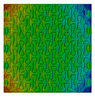	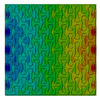	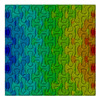	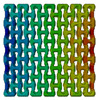
Transversal orientation of structure	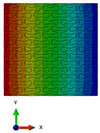	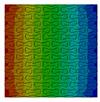	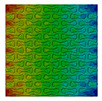	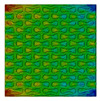	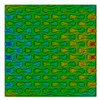	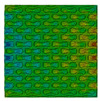	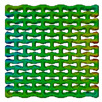
	Non-auxetic			ν≈0	Auxetic		

**Table 3:** Strain distribution and Poisson’s ratio’s for axially oriented composite structures.

Modulus of Filler, MPa $E_{filler}/E_{lattice}$	$\mathrm{Strain}\;\varepsilon_{11}$		Poisson’s Ratios	Global Poisson’s Ratio
200 (0.1)	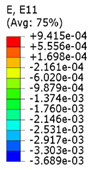	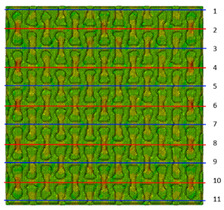	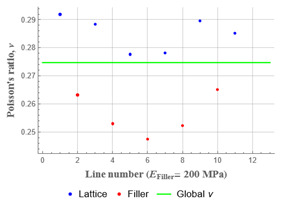	0.27
20 (0.01)	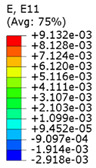	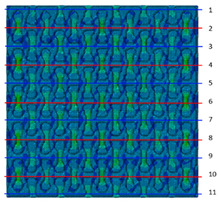	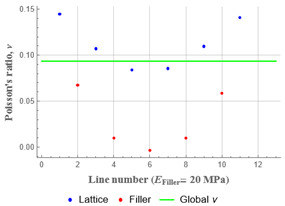	0.09
0.2 (10^−4^)	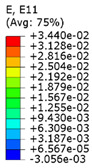	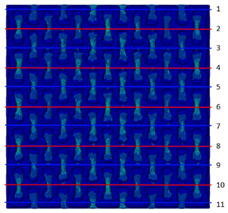	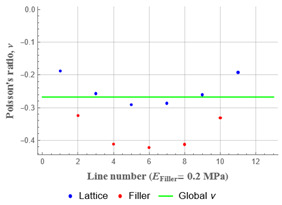	−0.27

**Table 4:** Strain distribution and Poisson’s ratio’s for transversely oriented composite structures.

Modulus of Filler, MPa $E_{filler}/E_{lattice}$	$\mathrm{Strain}\;\varepsilon_{11}$		Poisson’s Ratio	Global Poisson’s Ratio
200 (0.1)	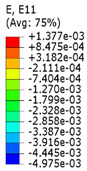	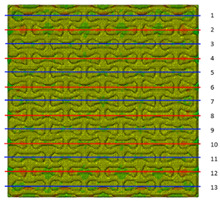	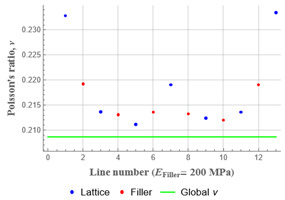	0.21
2 (10^−3^)	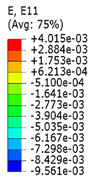	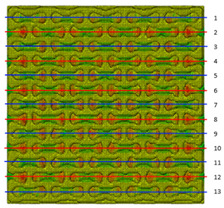	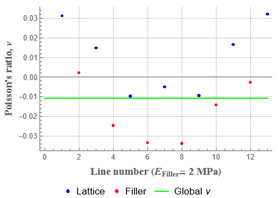	−0.01
0.2 (10^−4^)	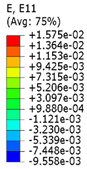	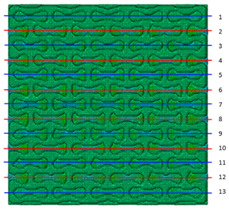	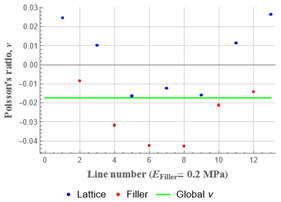	−0.02

## Data Availability

The data presented in this study are available on request from the corresponding author.
